# Mites Parasitic on Australasian and African Spiders Found in the Pet Trade; a Redescription of *Ljunghia pulleinei* Womersley

**DOI:** 10.1371/journal.pone.0039019

**Published:** 2012-06-13

**Authors:** Peter Masan, Christopher Simpson, M. Alejandra Perotti, Henk R. Braig

**Affiliations:** 1 School of Biological Sciences, Bangor University, Bangor, Wales, United Kingdom; 2 Institute of Zoology, Slovak Academy of Sciences, Bratislava, Slovakia; 3 School of Biological Sciences, University of Reading, Reading, United Kingdom; Biodiversity Insitute of Ontario – University of Guelph, Canada

## Abstract

Parasitic mites associated with spiders are spreading world-wide through the trade in tarantulas and other pet species. *Ljunghia pulleinei* Womersley, a mesostigmatic laelapid mite originally found in association with the mygalomorph spider *Selenocosmia stirlingi* Hogg (Theraphosidae) in Australia, is redescribed and illustrated on the basis of specimens from the African theraphosid spider *Pterinochilus chordatus* (Gerstäcker) kept in captivity in the British Isles (Wales). The mite is known from older original descriptions of Womersley in 1956; the subsequent redescription of Domrow in 1975 seems to be questionable in conspecificity of treated specimens with the type material. Some inconsistencies in both descriptions are recognised here as intraspecific variability of the studied specimens. The genus *Arachnyssus* Ma, with species *A. guangxiensis* (type) and *A. huwenae*, is not considered to be a valid genus, and is included in synonymy with *Ljunghia* Oudemans. A new key to world species of the genus *Ljunghia* is provided.

## Introduction

Close inspection of spiders often reveals mites associated with various body parts. Although these associations are most frequently reported from tropical spider families, mites are not uncommon on temperate spider species. Deutonymphs of Astigmata mites and Heterostigmata mites can be found phoretic on spiders; larvae of the Prostigmata families Erythraedae, Trombiculidae and Trombidiidae (chigger mites) can be parasitic on spiders, while Mesostigmata mites in the family Laelapidae often occur both as immature stages and adults on spiders [1]–[3]. Mites on spiders go back in time at least 50 Ma. Baltic amber shows phoretic and parasitic Acari together with jumping and cell spiders [4], [5]. In addition, free-living mites (Asigmata and Mesostigmata) can become a problem for captive tarantulas when high numbers start to occlude the moist surfaces of the book lungs [6]. The large number of saprophilous and predatory Mesostigmata might overshadow the host-specific associations particularly between spider and mites of the mesostigmatic family Laelapidae. However, specific associations are well documented. For example, all life stages of *Androlaelaps pilosus* Baker (Laelapidae) can be found on the hexathelid spider *Macrothele calpeiana* (Walckenaer), the only European tarantula [7]. Here we report laelapid mites living on captive *Pterinochilus chordatus* Gerstäcker, the Kilimanjaro mustard baboon spider.

The laelapid genus *Ljunghia* includes species that have established close associations with various mygalomorph spiders in Indonesia [8], [9], Malaysia [10], Australia [11]–[13], New Caledonia [14], Africa [15], and China [16]. It is assumed that they have developed obligatory parasitic relationships with their hosts [1]. To date, there is only one comprehensive review of *Ljunghia*, which includes a description of a new species from a Central American mygalomorph spider kept in captivity in Spain, a key for their identification and an enumeration of their host species [17].

Although there is no published record of an *Ljunghia* species from the British Isles, reports of mites parasitizing captive spiders is a common occurrence, often owing to contamination [17]. The presence of a seemingly Australian mite species on an African spider on the territory of the UK is interesting and might be either a consequence of the brisk business of tarantulas as pets including the wide-spread exchange of spiders among the breeders or an indication for a wider geographical distribution of *Ljunghia*.

The main aim of this study was a morphological redescription of *Ljunghia pulleinei*. Detailed observations of the most important morphological features of this mite allowed to discern more details than those reported in the original descriptions [11]. Generally, the original description of Womersley does not include illustrations of diagnostic morphologies as well as important metric data of some idiosomal structures and setae. There is one redescription of this species, that of Domrow [12], based on specimens that differ in some characters, e.g. distinctly shorter idiosomal setae when compared with the type specimens. Inconsistencies in the descriptions of Womersley and Domrow are another good reason for the following redescription.

## Results and Discussion

### Genus *Ljunghia* Oudemans

*Ljunghia* Oudemans, 1932: 204 [8]. Type species *Ljunghia selenocosmiae* Oudemans, 1932; by monotypy [8].

*Ljunghia (Metaljunghia)* Fain, 1989: 158 [9]. Type species *Ljunghia rainbowi* Domrow, 1975; by original designation [12].

*Arachnyssus* Ma, 2002: 8 [16]. Type species *Ljunghia guangxiensis* Ma, 2002; by original designation [16]. New synonymy.

#### Diagnosis (Adults)

Chelicerae chelate-dentate in female, with fixed digit usually reduced in size; cheliceral digits of male subequal in length, with curved spermatodactyl slightly exceeding the tip of the movable digit. Dorsal shield entire, not covering the whole dorsal surface, and with hypotrichous setation (at most, 32 pairs of setae present). Sternal shield with three pairs of setae, metasternal shields and setae often absent. A pair of genital setae present or absent, usually placed on epigynal shield. Anal shield relatively small, elongate, bearing three circum-anal setae. Leg setation holotrichous to markedly hypotrichous.

#### Notes on the genus

The genus *Ljunghia* was proposed by Oudemans [8], based on adults and deutonymphs collected from the theraphosid spider of the genus *Selenocosmia* Ausserer in Sumatra. Oudeman's genus *Ljunghia* has gained broad acceptance [9], [12], [15], [17], [20], mostly as a member of the subfamily Iphiopsinae within the family Laelapidae, and is currently divided into two subgenera, *Ljunghia* and *Metaljunghia*. We agree with Moraza *et al.* that this subgeneric structure is not useful, and it is not used here [17].

A new separate genus *Arachnyssus* was erected [16] to accommodate two mesostigmatic mite species associated with the mygalomorph theraphosid spider *Selenocosmia huwena* Wang, Peng & Xie ( = *Haplopelma schmidti* von Wirth, based on the newest taxonomic revision [21]) in China. The most important features that define the genus *Arachnyssus*, classified within the family Macronyssidae, are: (1) entire dorsal shield; (2) idiosomal setae very long, with tips reaching far beyond the insertions of following setae; (3) anus with anterior position to adanal setae; (4) coxae I–IV not armed with spines; (5) sternal shield saddle-shaped, with posterior margin deeply concave; (6) epigynial shield short, tongue-shaped; (7) anal shield drop-shaped; (8) epigynial and anal shields well separated [16].

It is obvious that the author who erected *Arachnyssus* neglected the existence of the genus *Ljunghia* because there is no reference to this genus in his paper [16] and all of the above characters enumerated for *Arachnyssus* can be found in *Ljunghia* [17]. Therefore *Arachnyssus* is here regarded as synonymous with *Ljunghia*, and the two species, namely *A. guangxiensis* and *A. huwenae* are therefore, newly transferred to the genus *Ljunghia*.

### *Ljunghia pulleinei* Womersley

*Ljunghia pulleini* Womersley, 1956: 591–593 [11].

*Ljunghia pulleini –* Domrow, 1975: 35–37 (in part: only specimens of the type series) [12].

*Ljunghia pulleinei* (emend. nov.) s. str. *–* Fain, 1991: 78–79 [13].

*Ljunghia* (*Metaljunghia*) *pulleini –* Fain, 1989: 159 [9]; Moraza *et al.*, 2009: 125 (in part) [17].

#### Material examined

4 females, 2 males – on *Pterinochilus chordatus* (det. R. C. Gallon) kept in captivity in the Laboratory of Molecular Parasitology, School of Biological Sciences, Bangor University, Gwynedd, NW Wales. The mites were collected by one of the authors, MAP, following the technique described aboved; October 2006.

#### Description (Adults)

**Figure 1:**
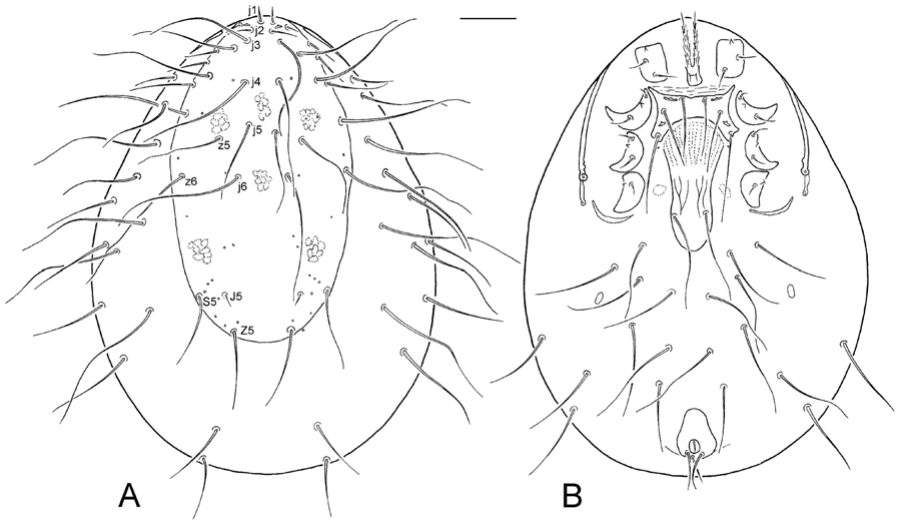
*Ljunghia pulleinei*, female. A, dorsal idiosoma (with setal notation of some dorsal setae); B, ventral idiosoma. Scale: 100 µm.

Female. Dorsal idiosoma (Figure 1A). Idiosoma oblong, egg-shaped, 810–860 µm long and 610–635 µm wide (650 µm long and 443 µm wide in freshly moulted and poorly sclerotized specimen). Dorsal shield entire, oblong, suboval, 560–595 µm long and 320–355 µm wide nearly at level of setae z5, usually not completely covering dorsal surface, with regularly rounded posterior margin and smooth surface. The shield free of anterior sections of peritremes, anterior ends of peritremes reaching close to paravertical setae z1. Podonotal region of the shield with 15 pairs of setae (j1–j6, z1, z2, z4–z6, s1–s4), opisthonotum with reduced complement of three setal pairs (J4, Z4 and Z5). Most dorsal shield setae simple, smooth, needle-like, sinuous and considerably elongated, the longest setae up to 270 µm in length and with thread-like distal part reaching far beyond the insertions of following setae; only setae j1, z1 and J4 short. Metric data for some selected dorsal setae as follows: j1 33–44 µm, j4 220–230 µm, j5 136–153 µm, j6 230–260 µm, J4 25–31 µm, z5 170–190 µm, Z4 142–152 µm, Z5 152–162 µm, the longest setae situated on soft membranous dorsal integument 220–255 µm. Dorsolateral membranous integument with 13 pairs of setae.

Ventral idiosoma (Figure 1B). Presternal platelets absent. Sternal shield almost quadrangular, longitudinally narrowed, 30–40 µm long in midline, 120–132 µm wide at level of setae st2 and 149–158 µm at level of setae st3, smooth on surface, deeply concave posteriorly; anterolateral corners well developed, slender and obtusely acuminate; the shield bearing two pairs of lyrifissures and three pairs of setae, length of sternal shield setae slightly increasing posteriorly: st1 100–115 µm, st2 105–122 µm, st3 150–170 µm. Metasternal platelets and setae st4 absent, a pair of metasternal lyrifissures placed on soft membrane close to posterolateral corners of sternal shield. Endopodal sclerites absent. Epigynal shield tongue-shaped, elongated, slightly constricted between coxae IV, hyaline anteriorly, rounded posteriorly, 238–252 µm long, 75–83 µm wide at level of genital setae, with a pair of genital setae st5 (166–184 µm) inserted in posterior part and a pattern of weak longitudinal lines on medial surface; associate genital pores not detected. Peritrematal shields almost fully reduced, only short and narrow poststigmatic section present; peritremes well developed, long and with stigma between coxae III and IV. Exopodal sclerites absent, parapodal sclerites developed, crescent. A pair of small and suboval metapodal platelets present. Anal shield pear-shaped, rounded anteriorly and posteriorly, 74–82 µm wide, smooth, bearing rounded anus and three circum-anal setae; postanal seta (64–77 µm) shorter than adanals (80–90 µm); anus with posterior position on the shield. Ventral and ventrolateral membranous integument with 10 pairs of setae. All ventral setae similar to those on dorsal idiosoma.

**Figure 2:**
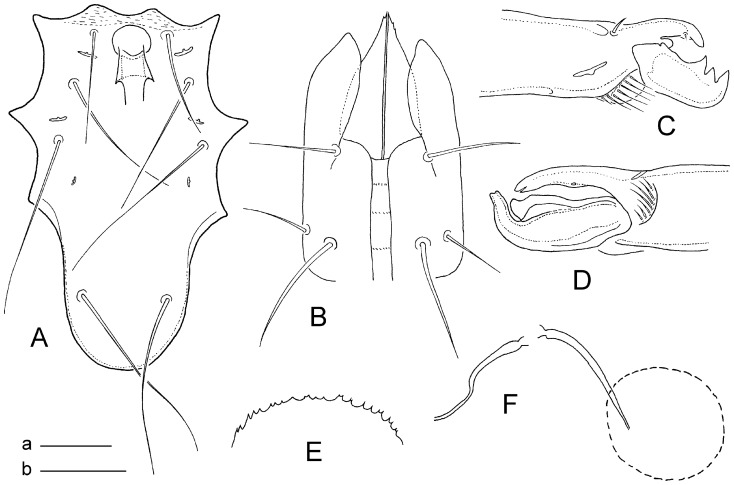
*Ljunghia pulleinei*. A, sternogenital shield of male; B, ventral hypostome of female (anterior part); C, cheliceral digits of female; D, cheliceral digits of male; E, epistome of female; F, tubular structures of insemination apparatus associated with coxae IV in female. Scales: a = 50 µm (Figs 2A, 2F), b = 25 µm (Figs 2B–E).

Gnathosomal structures (Figures 2B, 2C, 2E). Anterior ventral part of hypostome as in Figure 2B, with three pairs of simple hypostomal setae h1–h3; posterior setae h3 longest; posterior surface bearing a pair of simple postcoxal setae. Deutosternal groove relatively narrow and difficult to examine posteriorly, with only three detectable transverse rows of denticles on its anterior section. Corniculi obscure and covered by hypertrophied, lobe-like projection. Chelicerae chelate-dentate (Figure 2C); fixed digit reduced in size, markedly shorter and thiner than movable digit, and armed with distal hook; movable digit relatively robust, with distal hook and two massive subdistal teeth. Epistome rounded and serrate on anterior margin (Figure 2D).

Legs. All legs with a well developed pretarsus and ambulacral apparatus (including pulvillus and two claws), shorter than idiosoma. Leg segments without specific projections or macrosetae, with the chaetotactic pattern as previously described. Coxae IV associated with relatively thin and long tubular structures of insemination apparatus (Figure 2F).

Male (Figures 2A, 2D). Idiosoma 540–590 µm long and 360–395 µm wide, dorsal shield 490–515 µm long and 285–325 µm wide. Dorsum with a compact holodorsal shield. Metric data for some selected dorsal setae as follows: j1 23–29 µm, j3 177 µm, j4 200 µm, j5 110–115 µm, J4 12–19 µm, z5 134–158 µm, z6 184–207 µm, Z4 120–126 µm, Z5 126–132 µm. Venter with separate sternogenital (Figure 2A), and anal shields. Sternogenital shield oblong, subtruncate anteriorly, rounded posteriorly, 250–270 µm long in midline, 130–138 µm wide at level of setae st3 and 94–99 µm at level of setae st5, smooth on surface; the shield bearing three pairs of lyrifissures and four pairs of setae (st1–st3, st5), length of sternogenital shield setae slightly increasing posteriorly: st1 75–81 µm, st2 99–105 µm, st3 122–141 µm, st5 150 µm. Cheliceral digits subequal in length, without striking dentation; spermatodactyl hook-like, robust in basal part, curved distally (Figure 2D). Other characters almost identical as in female, including those on opisthogastric region.

#### Taxonomic notes

The original description of *Ljunghia pulleinei* was inadequately illustrated, the description itself was insufficient [11]; therefore, amendments followed in the redescription of Domrow [12], especially in the dorsal shield setation. For example, Womersley stated only 14 pairs of setae on the dorsal shield instead of 17–18 pairs documented by Domrow who examined three series of specimens: (1) the type material collected from theraphosid spider *Selenocosmia stirlingi* Hogg in South Australia; (2) specimens from a nemesiid spider of the genus *Aname* L. Koch found in South Australia; (3) specimens from an unidentified diplurid spider in Queensland. All three series were keyed out together by Domrow and, despite the presence of some morphological differences, indicative of a mixture of three species, they were declared to be conspecific [12]. We now know that the specimens from the spiders of the genus *Aname*, which differ from the typical series mainly by the lack of the subterminal pair of setae on the dorsal shield, belong to the species *Ljunghia aname*, which was originally described as a new subspecies of *L. pulleinei* [13], [14]. A third unknown species is being described by Bruce Halliday (personal communication).

Unfortunately, the description and figures of adults given by Domrow [12] and of deutonymphs of *Ljunghia pulleinei* by Fain [14]) did not apply to the mites of the type series but to those of an unknown diplurid spider. The type specimens and specimens introduced by Domrow (and Fain) can be easily distinguished from each other by the length of setae situated on medial surface of the dorsal shield. They belong to two species, and show a certain degree of interspecific variability not only in the length of idiosomal setae but also in position of some dorsal shield setae (especially in J4 and Z4). So, two main patterns of chaetotaxy can be distinguished: (1) type species with longer idiosomal setae, e.g. setae j5 and z5 with tips reaching far beyond the insertions of j6, setae j6 beyond the insertions of J4, and setae z5 beyond the lateral margin of dorsal shield; (2) species illustrated by Domrow and Fain with shorter idiosomal setae, e.g. setae j5 and z5 with tips not reaching the insertions of j6, setae j6 hardly reaching to the insertions of J4, and setae z5 clearly not reaching the lateral margin of dorsal shield.

*Ljunghia pulleinei* bearing the longer setae cannot be reliably identified in the newest key of the genus [17]; where some statements are solely applicable to the form exhibiting short setae as described by Domrow [12]. In addition there is a mistake in their key in relation to both setal forms: “setae j4 do not reach the tips of j6”. With exception of this inaccuracy and a pair of additional setae present on the opisthogastric ventral surface in our individuals, our description generally agrees with most of the published morphological characters given by Womersley and Domrow [11], [12]. In addition, we have included in our redescription new metric data for some idiosomal setae, and the shields.

*Ljunghia pulleinei* s. str. has here been collected from the Kilimanjaro mustard baboon spider from East Africa and previously from the common whistling spider from Australia. This is the first time that one and the same *Ljunghia* species has been associated with two different host species suggesting that *Ljunghia* species are not strictly species-specific. *Ljunghia* is well known from mygalomorph spiders but has also been reported from a more primitive liphistiid Malaysian trap door spider belonging to the Mesothelae [10]. Recently, new *Ljunghia* species have been retrieved from more liphistiid spiders from Vietnam and Thailand [22]. The female spiders showed clear bite marks of the mites on their prosomata emphasising the parasitic nature of *Ljunghia*.

### Key to the species now known in *Ljunghia* Oudemans

1. Opisthonotal region of dorsal shield with strongly suppressed setation, only 2–5 pairs of setae present 2
  - Setation of opisthonotal region moderately suppressed, 7–14 pairs of setae present 7
2. Opisthonotal region with two pairs of setae, podonotal region with 14 pairs of setae *Ljunghia aname* Fain, 1991
  - Opisthonotal region with at least three pairs of setae 3
3. Genital setae (st5) absent; podonotal region with 20 pairs of setae, male sternogenital shield with three pairs of setae (st1–st3) *Ljunghia novaecaledoniae* Fain, 1991
  - Genital setae present; podonotal region with at most 18 pairs of setae, male sternogenital or sternogenito-ventral shield with at least four pairs of setae 4
4. Podonotal region of dorsal shield with strongly suppressed setation, only 11 pairs of setae present, opisthonotal region with 4–5 pairs of setae 5
  - Setation of podonotal region moderately suppressed, 17–18 pairs of setae present, opisthonotal region with 3 pairs of setae 6
5. Opisthonotal region with 5 pairs of setae, setae J4 absent *Ljunghia bristowi* (Finnegan, 1933)
  - Opisthonotal region with 4 pairs of setae, setae J4 present *Ljunghia rainbowi* Domrow, 1975
6. Podonotal region with 14 pairs of setae, setae j5 absent; setae j6 and z5 relatively short and subequal in length *Ljunghia africana* Fain, 1991
  - Podonotal region with 15 pairs of setae, setae j5 present; setae j6 relatively long, obviously longer than setae z5 *Ljunghia pulleinei* Womersley, 1956
7. Metasternal setae absent; opisthonotal region with 8 pairs of setae 8
  - Metasternal setae present; opisthonotal region with at least 11 pairs of setae 9
8. Podonotal region with 17 pairs of setae; setae J4 present, minute; sternal shield subrectangular; male with sternogenital shield *Ljunghia hoggi* Domrow, 1975
  - Podonotal region with 20 pairs of setae; setae J4 absent; sternal shield saddle-like, deeply concave posteriorly; male with holoventral shield *Ljunghia guangxiensis* (Ma, 2002) comb. nov.
9. Opisthonotal region with 14 pairs of setae; male with sternogenital shield bearing five pairs of setae (st1–st5) *Ljunghia minor* Fain, 1989
  - Opisthonotal region with 11 pairs of setae; male with sternogenital-ventral or holoventral shield bearing five pairs of setae together with a number of additional ventral setae 10
10. Setae J4 not modified, subequal with most of other dorsal setae; male with sternogenital-ventral shield, ventral part of the shield with 5–7 setae *Ljunghia selenocosmiae* Oudemans, 1932
  - Setae J4 reduced in length, conspicuously shorter than other dorsal setae; male with holoventral shield bearing at least 13 setae in ventral part 11
11. Podonotal region with 21 pairs of setae; setae J4 shorter, with tips not reaching the posterior margin of dorsal shield; holoventral shield of male with 20–21 setae in ventral part *Ljunghia luciae* Moraza, Iraola & Alemany, 2009
  - Podonotal region with 20 pairs of setae; setae J4 longer, with tips reaching beyond the posterior margin of dorsal shield; holoventral shield of male with about 13 setae in ventral part *Ljunghia huwenae* (Ma, 2002) comb. nov.

## Materials and Methods

All examined specimens of *Ljunghia pulleinei* were obtained from the mygalomorph spider *Pterinochilus chordatus* kept in captivity in Gwynedd, North Wales. *P. chordatus* is an East African theraphosid species distributed in Ethiopia, Kenya, Somalia, Sudan, Tanzania and Uganda [18].

The collection of mites was carried out on the living spider. Due to the aggressivity of the spider it was necessary to develop a technique to collect the mites attached to the dorsal parts of the cephalothorax and the abdomen. The spider was reared in a plastic container covered by a plastic lid. Small holes of 2 mm where made in the lid to allow breading but also to allow insertion of a wire to touch the mites. The tip of the wire was soaked in 100% glycerol (glycerin) and then directed inside the cage towards every single mite. Due to the sticky nature of glycerol, the mites were instantly glued to the tiny tip of the wire and extracted from the cage by slowly moving the tip out to avoid distressing the spider. Once outside the cage, the tip of the wire with a glued mite was submerged in 96% ethanol where the mites detached and became fixed and preserved for further analysis.

The mites were mounted on permanent microscope slides using Swan medium. Illustrations were made by using a high magnification microscope equipped with a drawing tube. Measurements were made from slide-mounted specimens with stage-calibrated ocular micrometers. Lengths of shields and leg segments were measured along their midlines, and widths were measured at the widest point. Dorsal setae were measured from the bases of their insertions to their tips. Measurements are mostly presented as ranges (minimum to maximum). The terminology of dorsal and ventral chaetotaxy follows Lindquist & Evans [19]. For the specific chaetotactic notation of some dorsal shield setae, see Figure 1A. The redescribed specimens are deposited at the Institute of Zoology, Slovak Academy of Sciences, Bratislava, and the Australian National Insect Collection, Canberra, Australia (2♀♀, 1♂).
